# Disparities in transvenous lead extraction in young adults

**DOI:** 10.1038/s41598-022-13769-y

**Published:** 2022-06-10

**Authors:** Andrzej Kutarski, Wojciech Jacheć, Łukasz Tułecki, Marek Czajkowski, Dorota Nowosielecka, Paweł Stefańczyk, Konrad Tomków, Anna Polewczyk

**Affiliations:** 1 Department of Cardiology, Medical University of Lublin, Lublin, Poland; 2 2nd Department of Cardiology, Faculty of Medical Sciences in Zabrze, Medical University of Silesia in Katowice, Katowice, Poland; 3 Department of Cardiac Surgery, The Pope John Paul II Province Hospital, Zamość, Poland; 4 Department. of Cardiac Surgery, Medical University of Lublin, Lublin, Poland; 5 Department of Cardiology, The Pope John Paul II Province Hospital, Zamość, Poland; 6 Department of Physiology, Pathophysiology and Clinical Immunology, Collegium Medicum of Jan Kochanowski University, Kielce, Poland; 7 Department of Cardiac Surgery, Świętokrzyskie Center of Cardiology, Grunwaldzka Str. 45, 25-726 Kielce, Poland

**Keywords:** Cardiology, Medical research, Risk factors

## Abstract

Adults with cardiac implantable electronic devices (CIEDs) implanted at an early age constitute a specific group of patients undergoing transvenous lead extraction (TLE). The aim of this study is to assess safety and effectiveness of TLE in young adults. A comparative analysis of two groups of patients undergoing transvenous lead extraction was performed: 126 adults who were 19–29 years old at their first CIED implantation (early adulthood) and 2659 adults who were > 40 years of age at first CIED implantation and < 80 years of age at the time of TLE (middle-age/older adulthood). CIED-dependent risk factors were more common in young adults, especially longer implant duration (169.7 vs. 94.0 months). Moreover younger age of patients at first implantation, regardless of the dwell lead time, is a factor contributing to the greater development of connective tissue proliferation on the leads (OR 2.587; p < 0.001) and adhesions of the leads with the heart structures (OR 3.322; p < 0.001), which translates into worse TLE results in this group of patients. The complexity of procedures and major complications were more common in younger group (7.1 vs. 2.0%; p < 0.001), including hemopericardium (4.8 vs 1.3; p = 0.006) and TLE-induced tricuspid valve damage (3.2 vs.0.3%; p < 0.001). Among middle-aged/older adults, there were 7 periprocedural deaths: 6 related to the TLE procedure and one associated with indications for lead removal. No fatal complications of TLE were reported in young adults despite the above-mentioned differences (periprocedural mortality rate was comparable in study groups 0.3% vs 0.0%; p = 0.739). Predictors of TLE-associated major complications and procedure complexity were more likely in young adults compared with patients aged > 40 to < 80 years. In younger aged patients prolonged extraction duration and higher procedure complexity were combined with a greater need for second line tools. Both major and minor complications were more frequent in young adults, with hemopericardium and tricuspid valve damage being predominant.

## Introduction

Permanent cardiac pacing therapy (PM, ICD/CRT) is prescribed predominantly to older patients. Only a small proportion of cardiac implantable electronic devices (CIED) are implanted in subjects aged less than 40 years. Children and juveniles with leads implanted in childhood form a specific group of patients from the viewpoint of lead management and lead extraction in particular. Based on previous reports, the importance of rapid lead-induced tissue scarring and calcification has been emphasized^1–7^. The formation of this fibrous capsule in combination with somatic growth is a common source of mechanical lead damage and more difficult extraction in children than in adults^5–10^. To the best of our knowledge there is only one study addressing the peculiarities of lead extraction in young adults (< 40 years) compared with patients ≥ 40 years^10^. In the recent guidelines older age is considered as a risk factor for TLE-associated major complications and periprocedural mortality^11–13^. Life expectancy in young CIED carriers is long or very long and the guidelines recommend avoiding lead abandonment in this age group^11–13^.

The problem of lead extraction in young patients or older adults with CIED implanted in childhood is relatively unknown, and therefore requires detailed research. The purpose of the study was to compare effectiveness, complexity and major complications of transvenous lead extraction in patients aged 19–29 at the time of lead implantation and in subjects aged 40–80 at lead implantation/extraction. Patients with leads implanted in childhood and patients with TLE performed after the age of 80 were not included in this study because of age-specific differences.

## Methods

### Study population

This post-hoc analysis used clinical data of 3344 patients who underwent transvenous lead extraction between March, 2006 and September, 2020. All information relating to patients and procedures was entered into the computer on an ongoing basis. For the purposes of comparison the cohort was divided into two groups: group A consisting of 126 adult patients who were 19–29 years old, mean age 23.6 ± 3.1, at their first CIED implantation (mean age at extraction 37.9 ± 9.2) and group B comprised of 2659 adults who were > 40 years of age, mean age 58.4 ± 11.5, at the time of their CIED implantation and < 80 years of age, mean age 66.5 ± 9.4, at the time of transvenous lead extraction. No other patient exclusion criteria were used. Some patients with very old leads or abnormal lead route (strained, looped) were referred for elective system replacement at our tertiary reference care center.

This study analyzed demographic, clinical, CIED-related and procedure-related (including success and complications) data. The SAFeTY TLE score was used to predict the risk of major complications^14^, with an online calculator available at http://alamay2.linuxpl.info/kalkulator/.

### Lead extraction procedure

Lead extraction procedures were performed using mechanical systems such as polypropylene Byrd dilator sheaths (Cook® Medical, Leechburg, PA, USA), mainly via the implant vein. If technical difficulties arose, alternative venous approaches and/or additional tools such as Evolution (Cook® Medical, USA), TightRail (Spectranetix, USA) sheaths, lassos, basket catheters were utilized. Laser cutting sheaths were not used. In both groups lead extraction was performed by a team consisting of the same experienced operator, a second operator having experience with pacing therapy and a cardiac surgeon, whereas an anesthesiologist and echocardiographer were often but not always present during the procedure.

### Definitions

Indications for TLE and type of periprocedural complications were defined according to the 2017 HRS Expert Consensus Statement on Cardiovascular Implantable Electronic Device Lead Management and Extraction^12^.

Extraction procedures of lead(s) older than one year were defined according to the guidelines on management of lead-related complications (HRS 2009 and 2017, and EHRA 2018^11–13^.

Procedural success was defined as removal of all targeted leads and all lead material from the vascular space, without any permanently disabling complication or procedure-related death^11–13^.

Clinical success was defined as removal of all targeted leads or retention of a small (< 4 cm) portion of the lead that did not negatively impact the outcome goals of the procedure or permanently disabled the patient (only in patients with noninfectious indications for TLE)^11–13^.

Partial radiographic success was defined as leaving a lead tip or a fragment of lead less than 4 cm^11–13^.

### Statistical analysis

The Shapiro–Wilk test showed that most continuous variables were normally distributed. For uniformity, all continuous variables are presented as the mean ± standard deviation. The categorical variables are presented as numbers and percentages. The significance of differences between groups was determined using the nonparametric Chi^2^ test with Yates correction or because of the large disproportion in the size of the compared groups with the Mann–Whitney U test, as appropriate.

To determine which parameters have impact on the major complications (MC) occurrence and clinical and procedural success the following variables were included in the regression analysis of risk factors of major complication and prognostic factors of clinical and procedural success: patient’s age during the first CIED implantation, patients age during TLE, gender, value of left ventricle ejection fraction, cteatinine level, body mass index, Charlson’s comorbidity index, indications for TLE (infectious *vs* non-infectious), kind of CIED system (conventional, or with HV lead), presence/extraction of abandoned leads, number of leads in the heart (number of leads in the system + number of abandoned leads), number of CIED—related procedures before TLE and dwell time of the oldest extracted lead.

The variables with p < 0.1 in the one-variable regression analysis are presented in Table 5 and were included in the multivariate analysis. Due to the small number of major complications (n = 9), in group A, a two-variable analysis was performed comparing the dwell time of the oldest extracted leads with other variables, which achieved statistical significance (p < 0.1) under univariable analysis.

In order to assess the significance of the influence of the patient's young age during first implantation, and the dwell lead time a binary regression analysis was performed too. To analysis age of patients between 19 and 29 years during first CIED implantation and the dwell lead time above 10 years were included. Impact of above variables on the major complications occurrence, achieving of clinical and total procedural success, presence of connective tissue on the leads and connective tissue adhesions of leads to heart structures were tested.

Statistical analysis was performed with Statistica version 13.3 (TIBCO Software Inc.).

### Approval of the Bioethics Committee

All patients gave their informed written consent to undergo TLE. The use of anonymous data from patient’s medical records was approved by the Bioethics Committee at the Regional Chamber of Physicians in Lublin, Poland no. 288/2018/KB/VII.

All methods were performed in accordance with the relevant guidelines and regulations.

## Results

The mean difference in age at the time of lead extraction between group A (patients with lead implanted in early adulthood (ages 19–29) and B (patients with lead implanted at age > 40 and TLE performed before the age of 80) was 28.6 years (37.9 vs. 66.5). The mean difference in age at first lead/system implantation was 34.9 years (23.6 vs. 58.4). Compared with “ordinary” adults younger patients were more often women (46.8 vs. 37.4%). In young adults the most common indications for permanent cardiac pacing were congenital heart diseases, channelopathies, neurocardiogenic syncope and complications of cardiac surgery (82.5%), whereas in middle-aged/older adults ischemic heart disease, myocardial infarction (MI) and cardiomyopathies (77.5%). Age and indications for CIED are the indicators of difference in health status between the two groups, expressed as significant differences in average EF (59.2 vs. 47.8%), diabetes (2.4 vs. 21.1%), renal failure (3.2 vs. 20.4%) and Charlson comorbidity index (0.4 vs. 4.7). (Table 1).

**Table 1:** Clinical characteristics of the study population.

Groups of patients	Implantation ages 19–29		Implantation and TLE ages 40–80		A vs B
	A		B		Mann–Whitney U test, Chi^2^ P
Number of patients	126		2659		
Data for analysis	Count/average	%/SD	Count/average	%/SD	
Patient age during TLE (years)	37.85	9.22	66.49	9.38	P < 0.001
Patient age at first CIED implantation (years)	23.56	3.12	58.44	11.51	P < 0.001
Female	59	46.83%	994	37.38%	P = 0.041
Etiology of pacing: IHD, MI	7	5.56%	1636	61.53%	P < 0.001
Etiology of pacing: cardiomyopathy	15	11.90%	425	15.98%	P = 0.271
Etiology of pacing: congenital, channelopathies, neurocardiogenic, cardiac surgery	104	82.54%	598	22.49%	P < 0.001
Heart failure NYHA class III & IV	3	2.38%	401	15.08%	P < 0.001
Left ventricular ejection fraction [%]	59.20	9.80	47.81	15.48	P < 0.001
Left Ventricular Ejection Fraction < 41%	12	9.52%	929	34.94%	P < 0.001
Diabetes (any)	3	2.38%	561	21.10%	P < 0.001
Renal failure (any)	4	3.17%	543	20.42%	P < 0.001
Creatinine level [mg%]	0.94	0.79	1.24	1.84	P < 0.001
BMI (kg/m^2^)	25.89	4.35	28.31	5.40	P < 0.001
Long-term anticoagulation	23	18.25%	1076	40.47%	P < 0.001
Long-term antiplatelet treatment	10	7.94%	1229	46.22%	P < 0.001
Charlson comorbidity index	0.44	1.32	4.73	3.52	P < 0.001

*BMI* body mass index, *IHD* ischaemic heart diseases, *MI* myocardial infarction, *NYHA* New York Heart Association class.

Older patients were more likely to have infections compared with younger adults (32.3 vs. 21.4%), with the difference being significant for pocket infection (9.9 vs. 2.4%). Adults aged 19–29 were referred for lead extraction predominantly for noninfectious indications, mainly mechanical lead damage (46.0 vs. 25.7%). Middle-aged/older adults were more likely to have dysfunctional leads (12.5 vs. 5.6%) and system downgrading (0.8 vs. 3.8%), whereas younger aged patients more often underwent extraction of superfluous leads (10.3 vs. 3.2%). There were no differences in the type of CIED, excluding VDD (7.1 vs. 2.0%) and CRT-D (0.0 vs. 8.3%). Patients in group B tended to have more leads in the system than patients in group A (1.6 ± 0.5 vs. 1.8 ± 0.7 p = 0.003). Young adults had more, albeit insignificantly, abandoned leads (15.1 vs. 11.6%). Adults aged 19–29 were more likely to have more than 4 leads in the heart (3.2 vs. 0.4%), two single-coil ICD leads (8.1 vs. 0.6%), leads on both sides of the chest (5.6 vs. 3.0%) and more CIED-related procedures before lead extraction (2.4 vs. 1.9%) compared with patients aged 40–80. Young adults had significantly older leads: dwell time of the oldest extracted lead per patient was 172.1 months vs. 94.0 months in older adults and mean implant duration per patient before TLE was 152.3 vs. 86.8 months (Table 2).

**Table 2:** Analysis of risk factors for the difficulty of the procedure and major complications.

Groups of patients	Implantation ages 19–29		Implantation and TLE ages 40–80		A vs B
	A		B		Mann–Whitney U test, Chi^2^ P
Number of patients	126		2659		
Data for analysis	Count/average	%/SD	Count/average	%/SD	
**Noninfectious indications for TLE**					
Systemic infection	24	19.05%	595	22.38%	P = 0.442
Local (pocket) infection	3	2.38%	263	9.89%	P = 0.008
Mechanical lead damage (electric failure)	58	46.03%	682	25.65%	P < 0.001
Lead dysfunction (exit/entry block, dislodgement, extracardiac pacing)	7	5.56%	331	12.45%	P = 0.030
Other (perforation, upgrading, downgrading, abandoned lead, threatening/potentially threatening lead, MRI indication, cancer, painful pacemaker pocket, loss of indications for pacing, regaining venous access)	34	26.98%	786	29.56%	P = 0.963
**The main goal of TLE**					
System removal—infection	27	21.43%	858	32.27%	P = 0.014
Upgrading	19	15.08%	294	11.06%	P = 0.210
Downgrading	1	0.79%	100	3.76%	P < 0.001
Lead replacement	61	48.41%	1289	48.48%	P = 0.939
Superfluous lead extraction	13	10.32%	86	3.23%	P < 0.001
Other noninfectious indications	5	3.96%	32	1.20%	P = 0.020
**System and history of pacing**					
Pacemaker (any)	88	69.84%	1972	74.16%	P = 0.282
Pacemaker—VDD system	9	7.14%	53	1.99%	P < 0.001
ICD (VVI, DDD) pacing system	38	30.16%	638	34.93%	P = 0.141
ICD—CRT-D pacing system	0	0.00%	220	8.27%	P < 0.001
Number of leads in the system before TLE	1.63	0.50	1.83	0.70	P = 0.003
Presence of abandoned leads before TLE	19	15.08%	308	11.58%	P = 0.249
Multiple abandoned leads before TLE	7	5.56%	102	3.84%	P = 0.661
Number of leads in the heart before TLE	1.82	0.72	1.98	0.77	P = 0.037
4 and > 4 in the heart before TLE	4	3.17%	11	0.41%	P < 0.001
Two single-coil ICD leads before TLE	3	8.11%	15	0.56%	P = 0.056
CS lead before TLE	1	0.79%	481	18.09%	P < 0.001
Leads on both sides of the chest before TLE	7	5.56%	80	3.01%	P = 0.179
Previous TLE	8	6.35%	126	4.74%	P = 0.540
Upgrading or downgrading with lead abandonment	14	11.11%	163	6.13%	P = 0.040
Large lead loop on X-ray before TLE	11	8.80%	137	5.15%	P = 0.122
Number of procedures before lead extraction	2.38	1.30	1.87	0.07	P < 0.001
Dwell time of the oldest lead per patient before TLE	172.1	102.2	93.95	66.44	P < 0.001
Mean implant duration (per patient) before TLE	152.3	81.80	86.78	59.11	P < 0.001

*CRT* cardiac resynchronization therapy, *CS* coronary sinus, *DDD* dual chamber system, *ICD* implantable cardioverter defibrillator, *VVI* single chamber system, *VDD* single lead, dual chamber system, *TLE* transvenous lead extraction.

**Figure 1:**
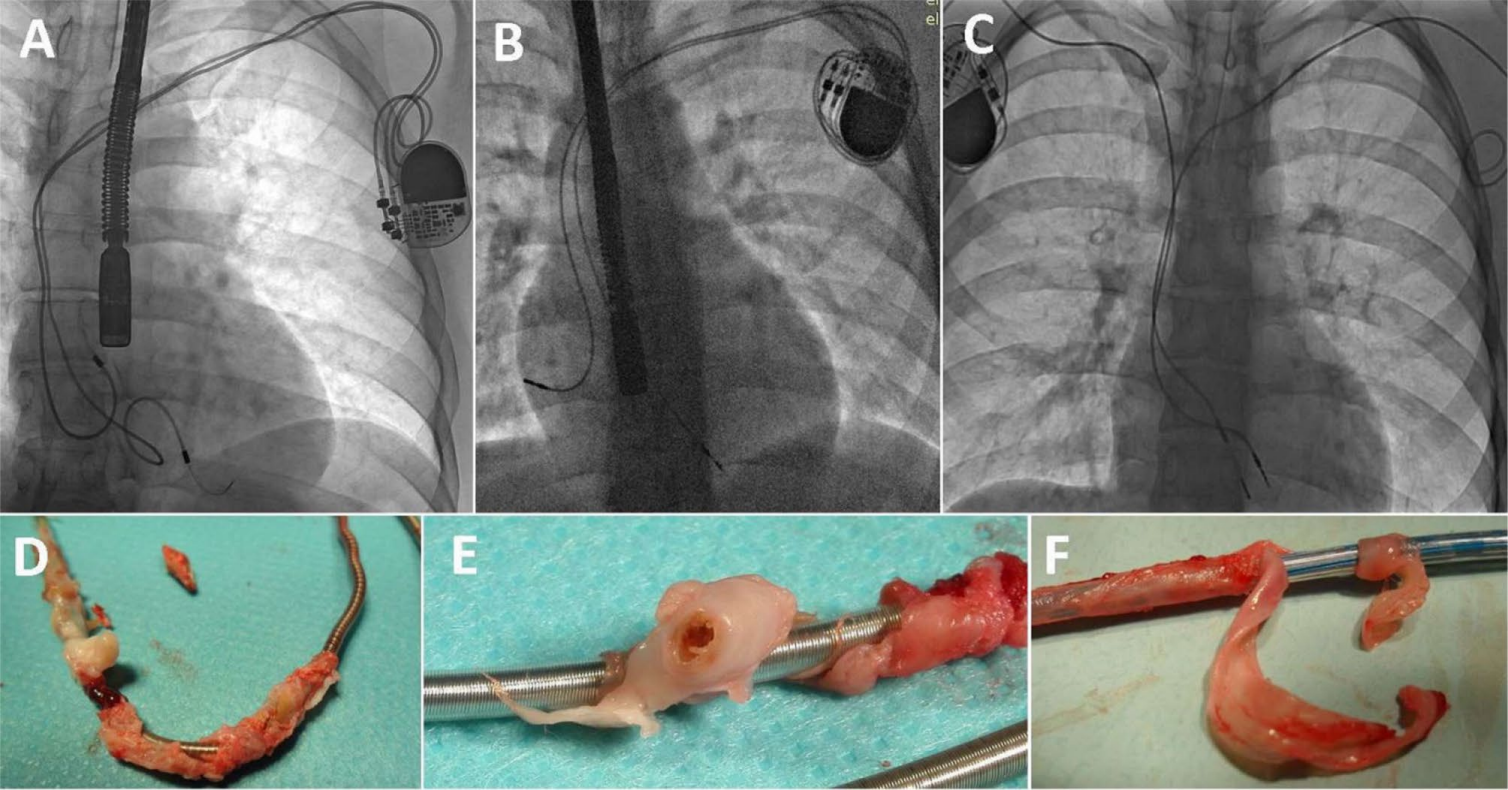
Several examples of X-ray and view of extracted leads. (**A**) Planned to long lead loops in the heart implanted 20 years before TLE. (**B**) Strained (“standing”) 12 y old dysfunctional RV lead due to body growth. RA lead was added 5 year ago during system upgrading. (**C**) 18-y old VVI pacing system (R) and abandoned 15-y old RV lead. (**D**) Strong advanced connecting tissue scar surrounding distal part of RAA lead. (**E**) Extracted in young adult ICD lead; strong massive scar (tunnel form) connecting two of leads before extraction—visible on one of two extracted leads. Such phenomenon make extraction more difficult. (**F**) Another form of (more floppy) scar on extracted ICD lead in young adult.

Extraction of VDD lead (10.3 vs 2.4%) and abandoned lead(s) (13.5 vs. 10.8%) were more frequent in younger than older adults. The average SAFeTY TLE score used to evaluate the risk of TLE-related major complications as the number of points^14^ was significantly higher in early adulthood (8.7 vs. 5.7 points). Lead extraction in early adulthood was more time and effort consuming: extraction duration, expressed as “skin-to-skin time” (66.9 vs. 60.2 min), “sheath-to-sheath time” (22.3 vs. 14.8 min), and mean extraction time per lead (sheath-to sheath/number of extracted leads) (14.1 vs. 8.7 min) was prolonged in younger adults. Occurrence of any technical problem during TLE (34.1 vs. 19.6%), Byrd dilator collapse/torsion/"fracture" (11.1 vs. 2.9%) and lead fracture/rupture during the extraction (14.3 vs. 5.6%) were significantly more common in younger adults. Lead-to-lead binding (9.5 vs. 6.8%) and the need to use alternative approach (14.7 vs. 1.6%) were markedly but not significantly (lead-to-lead binding) more frequent in early adulthood. Lasso catheters/snares for broken lead fragments were significantly more commonly used in younger patients (12.9 vs. 3.2%). Similarly, Evolution (old and new) or TightRail sheaths (2.4 vs. 1.1%), loops formed with the catheter, guide wire and lasso (to grasp the lead if the proximal end could not be reached) (2.4 vs. 1.8%) were more often required in young adults. Differences were not significant but a marked tendency was clear (Table 3) (Fig. 1).

**Table 3:** Detailed analysis of the risk factors associated with the procedure in terms of the complexity of the procedure and major complications.

System and procedure information	Implantation ages 19–29		Implantation and TLE ages 40–80		A vs. B
	A		B		Mann–Whitney U test, Chi^2^ P
Number of patients	126		2659		
Data for analysis	Count/average	%/SD	Count/average	%/SD	
**Potential risk factors for major complications and technical problems**					
Number of extracted leads per patient	1.74	0.87	1.67	0.77	P = 0.384
Need to use alternative approach	5	3.97%	84	3.16%	P = 0.806
Extraction of VDD lead	13	10.32%	64	2.41%	P < 0.001
Extraction of lead with too long loop	7	5.56%	94	3.54%	P = 0.387
Extraction of broken lead with too long loop	2	1.50%	67	0.00%	P = 0.715
Extraction of abandoned lead(s) (any)	17	13.49%	288	10.83%	P = 0.043
HV therapy (ICD) lead was extracted	38	30.16%	788	29.64%	P = 0.979
CS (LV pacing) lead was extracted	0	0.00%	185	6.96%	P = 0.004
Cumulative dwell times of extracted leads (in years)	22.60	16.90	13.16	12.57	P < 0.001
SAFETY TLE risk score (number of points)	8.70	5.00	5.65	4.21	P < 0.001
**TLE procedure duration**					
Procedure duration (skin to skin) (minutes)	66.90	35.46	60.18	25.87	P = 0.013
Procedure duration (sheath to sheath) (minutes)	22.27	32.77	14.81	22.75	P < 0.001
Mean extraction time per lead (sheath-to sheath/number of extracted leads) (minutes)	14.10	26.60	8.65	12.24	P < 0.001
**Procedure difficulty**					
Technical problem during TLE (any)	43	34.13%	521	19.59%	P < 0.001
Lead-to-lead binding	12	9.52%	181	6.81%	P = 0.320
Byrd dilator collapse/torsion/"fracture"	14	11.11%	77	2.90%	P < 0.001
Extracted lead fracture/rupture during extraction	18	14.29%	150	5.64%	P < 0.001
Loss of free lead fragment	1	0.79%	13	0.49%	P = 0.864
One technical problem only	27	21.43%	304	11.43%	P < 0.001
Two technical problems	11	8.73%	74	2.78%	P < 0.001
Need to use alternative approach	18	14.329%	43	1.62%	P < 0.001
**Use of additional tools**					
Evolution (old and new) or TightRail sheaths	3	2.38%	30	1.13%	P = 0.396
Metal sheaths	9	7.14%	183	6.88%	P = 0.947
Lasso catheter/snare	15	11.90%	86	3.23%	P < 0.001
Loop formed with catheter guide wire and lasso	3	2.38%	47	1.77%	P = 0.870
Temporary pacing during procedure	21	16.70%	267	10.04%	P = 0.025

*CS* coronary sinus, *HV* high voltage, *ICD* implantable cardioverter defibrillator, *LV* left ventricle, *TLE* transvenous lead extraction.

**Figure 2:**
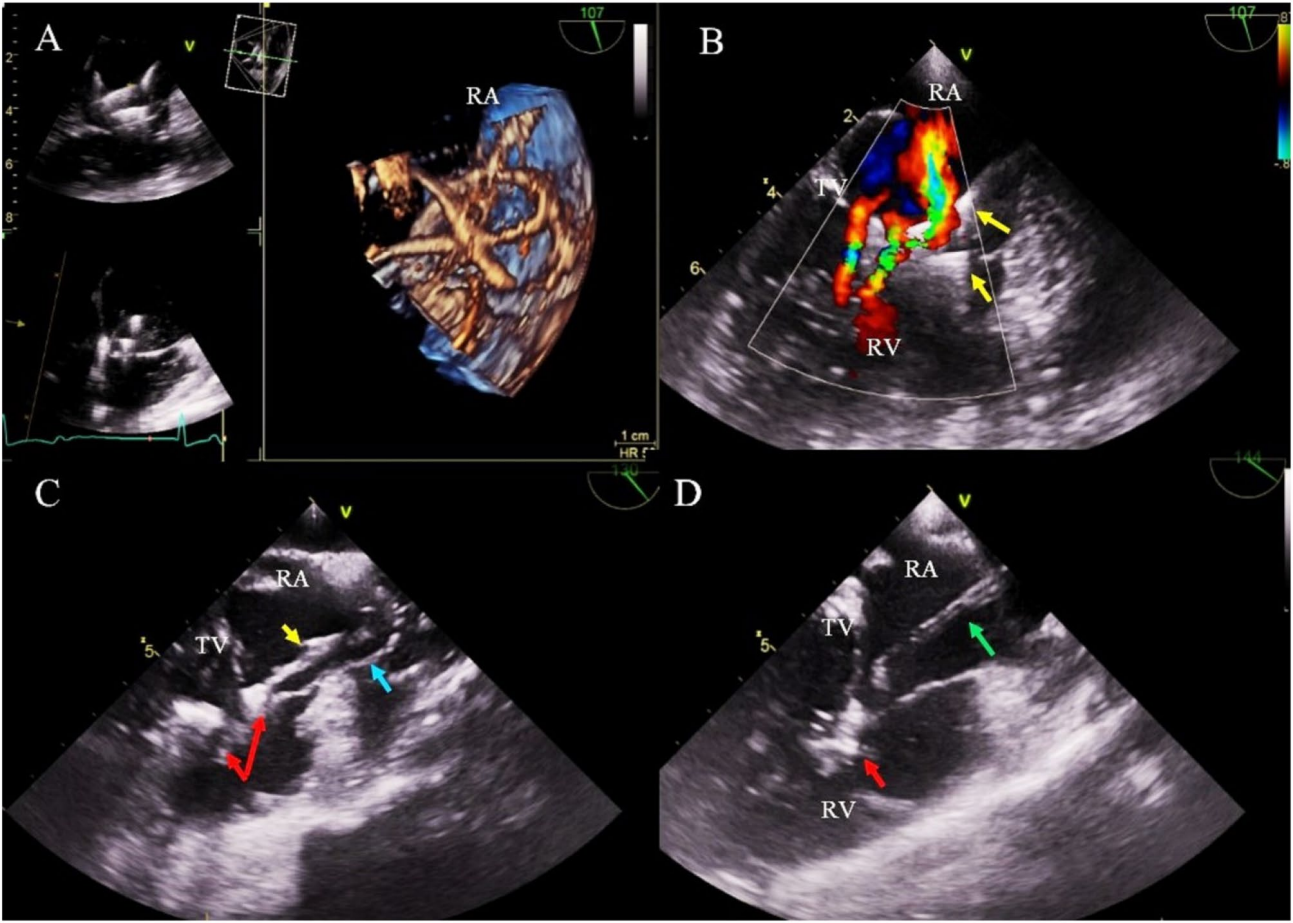
TEE images from the monitoring of the extraction of 4 leads in a 24-year-old female patient. (**A**) In the right atrium, loops of 4 leads fused together, displaced and adhered to the tricuspid apparatus. (**B**) Color Doppler—multi-flux, moderate tricuspid regurgitation with moderate valve stenosis (V max 1.6 m/s, PG avg. 4 mmHg) resulting from conglomerate of the leads (yellow arrows). (**C**) Moment of extraction of the lead; one of the ventricular leads is torn and stretched (blue arrow). Pulled up the second of the ventricular leads (yellow arrow) with simultaneous pull-up of the right ventricular wall and elements of the tricuspid apparatus. Red arrows mark massive adhesions of the leads with RV and TV structures. (**D**) After extraction of the leads, in the RA, a fragment of the silicone insulation was visualized by TEE examination (green arrow). Massive fragments of connective tissue within the sub-valvular apparatus (red arrow), hindering the proper mobility of the valve leaflets.

The organizational model plays an important role in extraction procedures. It does not influence the occurrence of major complications but strongly facilitates their proper management and reduces procedure-related mortality. Both groups underwent TLE in the same time interval (selection was done retrospectively). Between 2006 and 2011 extractions were performed in the EP-LAB, between 2012 and 2016 additional precautions (graded approach) were taken and since 2017 all procedures have been performed in the hybrid room or cardiac surgery operating room. Table 4 shows that young adults tended to undergo TLE in cardiac surgical operating rooms or hybrid rooms, with the cardiac surgeon as a co-operator, under general anesthesia and with TEE monitoring of lead extraction when possible (Fig. 2).

**Table 4:** Analysis of the effectiveness and complications of TLE.

Groups of patients	Implantation ages 19–29		I Implantation and TLE ages 40–80		A vs. B
	A		B		Mann–Whitney U test, Chi^2^ P
Number of patients	126		2659		
Data for analysis	Count/average	%/SD	Count/average	%/SD	
**Organizational model of TLE procedure**					
Procedure in cardiac surg. operating room or hybrid room	70	55.55%	1254	47.16%	P = 0.080
Cardiac surgeon as co-operator	72	57.10%	1262	47.46%	P = 0.042
General anesthesia	64	57.10%	1162	43.70%	P = 0.140
Routine TEE monitoring of lead extraction	59	46.80%	1022	38.44%	P = 0.073
Complete radiographic result (only X-ray)	113	89.68%	2564	96.43%	P < 0.001
Partial radiographic result (retained tip of lead)	5	3.97%	50	1.88%	P = 0.202
Partial radiographic result (retained < 4 cm lead fragment)	7	5.56%	40	1.50%	P = 0.003
Lack of radiographic result	1	0.79%	5	0.19%	P = 0.653
**Major complications**					
Major complications (all)	9	7.10%	54	2.03%	P < 0.001
Dwell time of extracted lead < 5 years	0/16	0.00%	6/1036	0.579%	P = 0.072
Dwell time of extracted lead 5–9 years	0/31	0.00%	9/937	0.961%	P = 0.384
Dwell time of extracted lead ≥ 10 years	9/79	11.39%	39/686	5.69%	P = 0.083
Hemopericardium	6	4.76%	35	1.32%	P = 0.006
Tricuspid valve damage during TLE (severe)	4	3.14%	9	0.34%	P < 0.001
Other major complications	0	0.00%	10	0.38%	P = 0.572
Rescue cardiac surgery	4	3.17%	33	1.24%	P = 0.150
Minor complications (any)	16	12.70%	201	7.56%	P = 0.053
Procedure-related death (intra-, post-procedural)	0	0.00%	6	0.23%	P = 0.653
Indication-related death (intra-, post-procedural	0	0.00%	1	0.08%	P = 0.876
**Radiographic success**					
Complete radiographic success (all material removed)	109	86.51%	2545	95.71%	P < 0.001
Dwell time of extracted lead < 5 years	16/16	100.0%	1021/1036	98.55%	P = 0.564
Dwell time of extracted lead 5–9 years	30/31	96.77%	904/937	96.48%	P = 0.684
Dwell time of extracted lead ≥ 10 years	63/79	79.75%	620/686	90.38%	P = 0.007
Partial radiographic success (retained tip or < 4 cm lead fragment)	13	10.32%	80	3.01%	P < 0.001
Lack of radiographic success (retained lead)	4	3.17%	34	1.28%	P = 0.004
**Clinical success**					
Clinical success	118	93.20%	2611	98.12%	P = 0.378
Dwell time of extracted lead < 5 years	16/16	100.0%	1031/1036	99.52%	P = 0.121
Dwell time of extracted lead 5–9 years	31/31	100.0%	926/937	98.83%	P = 0.893
Dwell time of extracted lead ≥ 10 years	71/79	89.87%	654/686	95.33%	P = 0.072
Lack of complete radiographic success in infectious cases	4	3.17%	28	1.28%	P = 0.973
TV damage	4	3.17%	9	0.34%	P < 0.001
Complication—death	0	0.00%	7	0.26%	P = 0.739
Planned cardiac surgery	0	0.00%	4	0.15%	P = 0.175
**Procedural success**					
Procedural success	109	86.51%	2545	95.71%	P < 0.001
Dwell time of extracted lead < 5 years	16/16	100.0%	1021/1036	98.55%	P = 0.564
Dwell time of extracted lead 5–9 years	30/31	96.77%	904/937	96.48%	P = 0.684
Dwell time of extracted lead ≥ 10 years	63/79	79.75%	620/686	90.38%	P = 0.007
Lack of complete radiographic success	13	10.32%	114	3.65%	P < 0.001
Permanently disabling complication or death	4	3.178%	18	0.68%	0. 023
**ECHO before and after TLE**					
Tricuspid regurgitation before TLE: moderate/severe	7	5.56%	362	13.61%	P = 0.013
Tricuspid regurgitation before TLE: severe	2	1.59%	89	3.35%	P = 0.407
**TLE related TV dysfunction (damage)**					
TR increase by 2 grades	9	7.14%	40	1.50%	P < 0.001
TR increase by 3 grades	2	1.59%	9	0.34%	P = 0.145

*TEE* transesophageal echocardiography, *TLE* transvenous lead extraction, *TV* tricuspid valve, *TR* tricuspid regurgitation.

Major complications were observed more frequently in younger than older adults (7.1 vs. 2.0%), similar to hemopericardium (4.8 vs. 1.3%), need for immediate cardiac surgery (3.2 vs. 1.2%) and severe tricuspid valve damage during TLE (3.2 vs. 0.5%). The differences were significant in all but one variable (immediate cardiac surgery), reflecting well the disparity in TLE safety between the two groups. The comparison of the proportion of major complications depending on lead dwell time showed significantly higher percentage of MC in young adults (with the leads above 10 years old). TLE effectiveness expressed as complete radiographic success (86.5 vs. 95.7) and partial radiographic success (10.3 vs. 3.0%), similar to clinical success (93.2 vs. 98.1%) and procedural success (86.5 vs. 95.7%) was worse in younger than older adults. The relatively low percentage of procedural success was caused mainly by the lack of complete radiographic success (10.3 vs. 3.0%) but also because of the occurrence of permanently disabling complications (3.2 vs. 1.3%). Lead dwell time, number of the leads, presence of abandoned leads and redundant lead loops crossing the tricuspid valve and the occurrence of TLE-related TV dysfunction (increased TR by 2 or 3 degrees) (8.7 vs. 1.8%) were also of more importance in younger than older adults. Clinical success in both groups was comparable, but the rates of complete radiographic and procedural success were lower in younger adults. The relatively low rate of procedural success in both groups was caused mainly by the lack of complete radiographic success and because of the occurrence of permanently disabling complications (TV damage). Procedural and clinical success in young adults with the oldest leads (above 10 years) was significantly lower (Table 4).

### Results of multivariable regression analysis

#### Major complications

In group of younger patients two-variable regression analysis shown that the only higher dwell time of the extracted lead was a risk factor of MC in this group. Probability of MC occurrence increased from 7.8 to 13.7% per one year of dwell time of the oldest extracted lead depending on compared data. The result of the juxtaposition in the two-variable analysis of the patient's age during TLE and the dwell time of the oldest extracted lead does not show statistical significance. This is due to the high correlation coefficient of both variables in this group (Spearman r = 0.888; p < 0.001).

In the group of older patients higher probability of MC occurrence was in patients with older leads—risk increase by 9.2% per one year (OR 1.092; p = 0.006), in female—risk increase by 236% (OR 3.361; p = 0.001) and in patients with higher number of previously CIED related procedures before TLE—risk increase by 30.3% per one procedure (OR 1.303; p = 0.071).

#### Clinical success

Multivariable regression analysis shown that in the group of younger patients only the number of previously CIED-related procedures had the impact on clinical success achieving, decreasing probability by 67.2% per one procedure (OR 0.328; p = 0.030). In the group of older patients lower probability of achieving of procedural success was in patients with older leads—reduction by 8.4% per one year (OR 0.916; p = 0.003), higher number of leads in the heart—reduction by 40.8% per one lead (OR 0.592; p = 0.014) and in the patients with infectious indications for TLE—reduction by 88.1% (OR 0.119; p < 0.001).

### Procedural success

Multivariable regression analysis shown that in the group of younger patients the TLE of conventional pacemaker leads decreased probability of achieving procedural success by 75.1% (OR 0.249; p = 0.025) and each previously CIED—related procedures by 41.4% (OR 0.586; p = 0.078), but this parameter achieving borderline statistically significance. In the group of older patients lower probability of achieving of procedural success was in patients with older leads—reduction by 7.1% per one year (OR 0.929; p < 0.001), higher number of previously CIED—related procedures—reduction by 19.1% per one procedure (OR 0.809; p = 0.031) and in the patients with the higher number of leads—reduction by 26.2% per each lead (OR 0.738; p = 0.056) (Table 5).

**Table 5:** Risk factors of major complication and prognostic factors of clinical and procedural success. Results of uni- and multi-variable regression.

Group A	Univariable model regression			Multivariable model regression		
	OR	95%CI	P	OR	95%CI	P
**Major complications**						
Patient age during TLE [year]	1.124	1.045–1.210	P < 0.001	1.053 ^(1)^	0.864–1.283	P = 0.605
Female	4.375	0.858–22.31	P = 0.073	3.862 ^(2)^	0.681–21.91	P = 0.123
Creatinine concentration [mg %]	0.021	0.000–1.505	P = 0.073	0.028 ^(3)^	0.003–2.596	P = 0.118
Extraction of abandoned lead	5.050	1.208–21.11	P = 0.025	1.449 ^(4)^	0.244–8.612	P = 0.680
Dwell time of the oldest extracted lead [year]	1.136	1.049–1.230	P = 0.002	1.078 ^(1)^ 1.137 ^(2)^ 1.136 ^(3)^ 1.124 ^(4)^	0.871–1.334 1.044–1.236 1.045–1.236 1.025–1.234	P = 0.485 P = 0.003 P = 0.002 P = 0.012

Group B	Univariable regression			Multivariable regression		
	OR	95%CI	P	OR	95%CI	P
Patient’s age at first CIED implantation [year]	0.949	0.916–0.983	P = 0.004	0.992	0.949–1.038	P = 0.740
Female gender	4.314	2.130–8.740	P < 0.001	3.361	1.618–6.984	P < 0.001
Extraction of HV lead	0.257	0.091–0.728	P = 0.011	0.561	0.186–1.687	P = 0.303
Presence of abandoned lead	4.001	2.016–7.942	P < 0.001	1.308	0.498–3.439	P = 0.586
Number of leads in the heart before TLE	1.670	1.166–2.392	P = 0.005	1.148	0.716–1.838	P = 0.567
Number of previously CIED- related procedures before TLE	1.770	1.469–2.132	P < 0.001	1.303	0.977–1.736	P = 0.071
Dwell time of the oldest extracted lead [year]	1.157	1.111–1.205	P < 0.001	1.092	1.026–1.162	P = 0.006

Group A	Univariable regression			Multivariable regression		
	OR	95%CI	P	OR	95%CI	P
**Clinical success**						
Patient’s age during TLE	0.922	0.859–0.990	P = 0.024	0.955	0.747–1.220	P = 0.708
Creatinine concentration [mg %]	48.91	0.535–4474	P = 0.088	3.137	0.019–521.1	P = 0.658
Infective TLE indications	0.253	0.058–1.099	P = 0.064	0.426	0.045–4.053	P = 0.453
Presence of abandoned leads	0.155	0.035–0.694	P = 0.014	0.342	0.011–10.70	P = 0.537
Number of the leads in the heart	0.359	0.155–0.834	P = 0.016	2.276	0.395–13.12	P = 0.353
Number of previously CIED related procedures	0.408	0.246–0.675	P < 0.001	0.328	0.119–0.907	P = 0.030
Dwell time of the oldest extracted lead [year]	0.923	0.856–0.955	P = 0.035	1.107	0.851–1.440	P = 0.443

Group B	Univariable regression			Multivariable regression		
	OR	95%CI	P	OR	95%CI	P
Patient’s age at first CIED implantation [year]	1.043	1.011–1.075	P = 0.008	1.015	0.978–1.054	P = 0.425
Left ventricle ejection fraction	0.978	0.959–0.998	P = 0.029	0.988	0.964–1.013	P = 0.359
Infectious indications for TLE	0.121	0.060–0.244	P < 0.001	0.119	0.057–0.250	P < 0.001
Extraction of HV lead	3.015	1.348–6.742	P = 0.007	1.706	0.646–4.505	P = 0.281
Presence of abandoned lead	0.286	0.153–0.534	P < 0.001	1.555	0.648–3.728	P = 0.322
Number of leads in the heart before TLE	0.495	0.366–0.669	P < 0.001	0.592	0.390–0.899	P = 0.014
Number of previously CIED—related procedures before TLE	0.564	0.476–0.668	P < 0.001	0.854	0.663–1.101	P = 0.223
Dwell time of the oldest extracted lead [year]	0.896	0.863–0.930	P < 0.001	0.916	0.864–0.971	P = 0.003

Group A	Univariable regression			Multivariable regression		
	OR	95%CI	P	OR	95%CI	P
**Procedural success**						
Patient’s age during TLE	0.936	0.888–0.986	P = 0.012	0.898	0.628–1.283	P = 0.551
Patient’s age at first CIED implantation	1.189	0.998–1.418	P = 0.051	1.353	0.888–2.064	P = 0.156
Pacemaker (AAI, VVI, DDD, VDD, CRT-P)	0.220	0.074–0.645	P = 0.006	0.249	0.073–0.854	P = 0.025
Number of previously CIED—related procedures before TLE	0.530	0.370–0.761	P < 0.001	0.586	0.321–1.068	P = 0.078
Dwell time of the oldest extracted lead [year]	0.914	0.863–0.968	P = 0.002	1.081	0.777–1.502	P = 0.642

Group B	Univariable regression			Multivariable regression		
	OR	95%CI	P	OR	95%CI	P
Patient’s age at first CIED implantation	1.045	1.022–1.068	P < 0.001	1.009	0.983–1.037	P = 0.491
Left ventricle ejection fraction [1%]	0.984	0.971–0.998	P = 0.025	0.992	0.977–1.007	P = 0.307
Extraction of HV lead	2.470	1.341–4.548	P = 0.004	1.339	0.676–2.653	P = 0.403
Presence of abandoned lead	0.310	0.195–0.492	P < 0.001	0.980	0.511–1.882	P = 0.953
Number of leads in the heart before TLE	0.573	0.455–0.721	P < 0.001	0.738	0.540–1.008	P = 0.056
Number of procedures before TLE	0.607	0.530–0.695	P < 0.001	0.809	0.668–0.981	P = 0.031
Dwell time of the oldest extracted lead [year]	0.892	0.867–0.917	P < 0.001	0.929	0.892–0.976	P < 0.001

*TLE* transvenous lead extraction, ^(1), (2), (3), (4)^—pairs of variables compared in the two-variable regression analysis, *CIED* cardiac implantable electronic device, *HV* defibrillating (high voltage) lead, *AAI* pacemaker system with the tip of lead in right atrium, *VVI* pacemaker system with the tip of lead in right ventricle, *DDD* dual chamber pacemaker system, *VDD* pacemaker system with the tip of integrated lead in right ventricle, *CRT-P* cardiac resynchronisation therapy pacemaker.

The binary regression analysis of the age of patients during the implantation with lead dwell time of the oldest lead in the patient showed that patient’s age during first CIED implantation between 19–29 years (group A) was an independent factor of the occurrence of major complications (OR 4.709; p < 0.001) and the lack of procedural success (OR 0.291; p = 0.002). Younger age of patients at first implantation, regardless of the dwell lead time, is also a factor contributing to the greater development of connective tissue proliferation on the leads (OR 2.587; p < 0.001) and adhesions of the leads with the heart structures (OR 3.322; p < 0.001), which translates into worse TLE results in this group of patients (Table 6).

**Table 6:** Binary analysis of impact of patients age during first CIED implantation and dwell time of oldest lead and oldest extracted lead on the major complication, clinical and total procedural success and connective tissue on the leads and connective tissue binding of the leads to the heart structures occurrence.

	Univariable regression analysis			Binary regression analysis		
	OR	95%CI	P	OR	95%CI	P
**Major complication**						
Patient's age during first system implantation: 19–29 vs 40–80 years	4.709	2.265–9.792	< 0.001	2.507	1.160–5.421	0.019
Dwell time of the oldest lead in the patient before TLE ≥ 10 years	11.203	5.388–23.30	< 0.001	10.035	4.776–21.09	< 0.001
Patient's age during first system implantation: 19–29 vs 40–80 years	4.709	2.265–9.792	< 0.001	2.548	1.177–5.515	0.018
Dwell time of the oldest extracted lead ≥ 10 years	10.454	5.170–21.14	< 0.001	9.344	4.569–19.11	< 0.001
**Clinical success**						
Patient's age during first system implantation: 19–29 vs 40–80	0.291	0.135–0.628	0.002	0.540	0.245–1.189	0.126
Dwell time of the oldest lead in the patient before TLE ≥ 10 years	0.147	0.082–0.264	< 0.001	0.157	0.087–0.284	< 0.001
Patient's age during first system implantation: 19–29 vs 40–80	0.291	0.135–0.628	0.002	0.540	0.245–1.189	0.126
Dwell time of the oldest extracted lead ≥ 10 years	0.164	0.093–0.288	< 0.001	0.157	0.087–0.284	< 0.001
**Procedural success**						
Patient's age during first system implantation: 19–29 vs 40–80	0.291	0.135–0.628	0.002	0.457	0.258–0.807	0.007
Dwell time of the oldest lead in the patient before TLE ≥ 10 years	0.147	0.082–0.264	< 0.001	0.191	0.128–0.287	< 0.001
Patient's age during first system implantation: 19–29 vs 40–80	0.291	0.135–0.628	0.002	0.446	0.252–0.790	0.006
Dwell time of the oldest extracted lead ≥ 10 years	0.164	0.093–0.288	< 0.001	0.203	0.136–0.302	< 0.001
**Fibrous binding of the lead to heart structures**						
Patient's age during first system implantation: 19–29 vs 40–80	3.322	2.136–5.166	< 0.001	2.073	1.305–3.293	0.002
Dwell time of the oldest lead in the patient before TLE ≥ 10 years	3.811	2.888–5.031	< 0.001	3.505	2.637–4.660	< 0.001
Patient's age during first system implantation: 19–29 vs 40–80	3.322	2.136–5.166	< 0.001	2.068	1.303–3.283	0.002
Dwell time of the oldest extracted lead ≥ 10 years	3.818	2.899–5.028	< 0.001	3.516	2.652–4.662	< 0.001
**Connective tissue on the leads (accretions. lead thickening. fibrous lead-lead binding)**						
Patient's age during first system implantation: 19–29 vs 40–80	2.587	1.762–3.798	< 0.001	1.828	1.225–2.728	0.003
Dwell time of oldest lead in the patient before TLE ≥ 10 years	2.400	1.960–2.938	< 0.001	2.255	1.833–2.774	< 0.001
Patient's age during first system implantation: 19–29 vs 40–80	2.587	1.762–3.798	< 0.001	1.864	1.251–2.777	0.002
Dwell time of the oldest extracted lead ≥ 10 years	2.458	2.008–3.009	< 0.001	2.308	1.876–2.838	< 0.001

## Discussion

Transvenous lead extraction is now an increasingly common procedure used in the therapy of CIED-related complications. The risk assessment of procedure is often difficult and misinterpreted. Knowledge of major complication predictors (long implant duration, female gender, renal failure, multiple leads, anemia, previous CIED-related procedures)^11–14^ in combination with risk factors for short- and long-term mortality after lead extraction (infections, old age, diabetes, low EF, heart failure)^11–13,15,16^ often leads to an unjustified overestimation of TLE-related risk.

There are only five reports on lead extraction in children^1–5^ and three reports on lead extraction in juveniles^6–9^, all of them confirming the difficulty of lead removal in these age groups^1–9^. There are also several reports on the expected increase in procedural risk but none of them has considered younger age of patients undergoing TLE as a risk factor for major complications^17–21^. Only one study uses the SAFeTY TLE score to assess the risk for the occurrence of major complications based on the following parameters: sum of dwell times of extracted leads (threshold value ≥ 16.5 years), hemoglobin concentration (threshold level < 11.5 g/dl), female gender, the number of previous CIED-related procedures and less than 30 years of age at first CIED implantation^14^. The TLE score calculator is an online tool available at http://alamay2.linuxpl.info/kalkulator/.

The current study shows that young adults were most likely to have congenital heart disease and complications of cardiac surgery. The majority of patients were in very good condition (Charlson comorbidity index 0.44). Noninfectious indications, especially mechanical lead damage were prevalent in young adults. There were no differences in CIED systems, but young adults had more abandoned leads and more leads on both sides of the chest and more previous CIED-related procedures. Currently, in accordance with the 2017 HRS consensus statement^12^, early removal of potentially dysfunctional leads should be considered in young people. As young patients have a very long life expectancy, abandonment of superfluous functional leads should be avoided, therefore the idea of prophylactic lead extraction needs further discussion.

Multivariable analysis of the risk of major complications in the current study revealed only a few common risk factors for MC in young and older adults. The most important finding in younger adults was longer dwell time of the oldest lead before TLE. As is well known, longer lead dwell time is the main risk factor for complications of TLE^12–14,19,20^. The present study confirmed that the effectiveness of the procedure was significantly lower in younger patients. Moreover, it has been shown that during lead extraction in young adults, technical problems occurred more frequently. Young adults more often needed the use of alternative approach and the second line tools (lasso catheters, Evolution or TightRail sheaths and loops formed with the catheter, guide wire and lasso). Mean extraction time per lead (sheath-to sheath/number of extracted leads) was longer in young adults (14.10 vs. 8.65 min). Major complications (including hemopericardium, immediate cardiac surgery and TLE-associated tricuspid valve damage) were also more common in young adults (7.10 vs. 2.03%). This finding is consistent with that of El-Chami who compared two groups: < 40 years of age (n = 84) and ≥ 40 (n = 690) and showed that the younger cohort more frequently tended to require the second line tools and techniques despite similar lead dwell times in both groups (5.7 vs. 5.6 years)^10^. A slightly different result in our study can be accounted for by different implant duration: 172.1 vs. 93.95 months (14.34 vs. 7.83 years) and a very specific group of young adults (ages 19–29 vs. > 40 years) who seem to be nearer to children in terms of scar formation. Similarly, the analysis of the effectiveness of TLE in the population of young adults with congenital heart disease showed slightly better results, however the age of the extracted leads was lower compared to the present study^21^. As the evidence shows patient age has a significant impact on the severity of scar formation, and in consequence, lead extraction difficulty^1–10^.

This study aims to remind that in such specific patients lead extraction should be performed by most experienced operators in a high-volume center, as previously suggested by El-Chami^10^.

Probably age at CIED implantation is a strong, but largely underestimated risk factor for major complications associated with transvenous lead extraction.

### Study limitations

There are some limitations in this study. Extractions were performed based on the organizational model evolving from graded safety precautions between 2006 and 2015 to full safety precautions since 2017. All types of mechanical systems but not laser powered sheaths were used. TLE was performed in three centers by the same very experienced first operator. The database was created prospectively, but analysis was performed retrospectively.

## Conclusions

Lead extraction in young adults is often more complicated compared to the older population, because younger aged patients have a more pronounced connective tissue reaction to the presence of the lead and a dwell time of the oldest lead before TLE is usually significantly longer. These factors have the greatest impact on the effectiveness and safety of the procedure. In patients with their leads implanted in early adulthood prolonged extraction duration and higher procedure complexity are combined with a greater need for second line tools and clinical and procedural success are lower due to the lack of complete radiographic success. Moreover, both major and minor complications are more frequent in young adults, with hemopericardium and tricuspid valve damage being predominant.

## Data Availability

All data generated or analysed during this study are included in this published article.
